# A Retrospective Study on the Relationship Between Calcar Femorale Injury and Postoperative Complications of Femoral Neck Fracture in Young and Middle‐Aged Patients Treated With Three Cannulated Screws

**DOI:** 10.1111/os.70147

**Published:** 2025-08-13

**Authors:** Qi Liu, Kaijun Zhang, Bin Zhao, Haoyuan Du, Teng Zhang, Wei Han, Junqiang Wang

**Affiliations:** ^1^ Department of Orthopaedics and Traumatology Beijing Jishuitan Hospital, Capital Medical University Beijing China; ^2^ National Center of Orthopaedics Beijing China; ^3^ Peking University Fourth School of Clinical Medicine Beijing China

**Keywords:** calcar femorale, femoral head necrosis, femoral neck fractures, femoral neck shortening, nonunion, postoperative complications

## Abstract

**Objectives:**

Given the critical biomechanical role of the calcar femorale in load transmission and fracture stability, understanding its relationship with postoperative complications is essential for optimizing surgical outcomes. Therefore, this study aimed to explore the relationship between calcar femorale injury and postoperative complications of femoral neck fracture in young and middle‐aged patients.

**Methods:**

A retrospective analysis was conducted on 350 femoral neck fracture patients (aged 18–65 years) treated with closed reduction and three cannulated screws fixation at a single institution from 2015 to 2020. Evaluate the clinical and imaging information of patients such as sex, age, body mass index, Garden classification, calcar femorale injury situation, computed tomography Hounsfield units (CT HUs), comorbidities (e.g., diabetes, hypertension etc.) and complications (femoral neck shortening, nonunion, and femoral head necrosis). CT‐based 3D reconstruction was used to analyze calcar femorale morphology. Statistical analyses included univariate and multivariate logistic regression to identify independent risk factors.

**Results:**

A total of 284 patients were included for analysis according to the inclusion and exclusion criteria. The results showed that Garden classification with displaced type (*p* < 0.001, OR = 4.615, 95% CI: 2.479–8.593), calcar femorale injury (*p* = 0.026, OR = 1.990, 95% CI: 1.087–3.645) and lower CT HUs (*p* = 0.002, OR = 0.989, 95% CI: 0.982–0.996) were independent risk factors for femoral neck shortening. Whether the patient has diabetes (*p* = 0.005, OR = 10.069, 95% CI: 2.043–49.628) was an independent risk factor for femoral neck nonunion. BMI (*p* = 0.030, OR = 1.154, 95% CI: 1.014–1.313) and Garden classification with displaced type (*p* < 0.001, OR = 10.000, 95% CI: 2.950–33.903) were independent risk factors for femoral head necrosis.

**Conclusion:**

This study found that older patients with displaced type femoral neck fractures with calcar femorale injury are more likely to experience femoral neck shortening. Clinicians should pay close attention to the above risk factors to reduce the incidence of postoperative complications in young and middle‐aged patients with femoral neck fractures.

## Graphical abstract

By highlighting the significance of calcar femorale injury and other risk factors, our findings can guide clinical doctors to closely monitor patients or design better internal fixation devices and surgical methods (such as DHS with anti‐rotational screw or medial buttress plating) to reduce the incidence of postoperative complications.

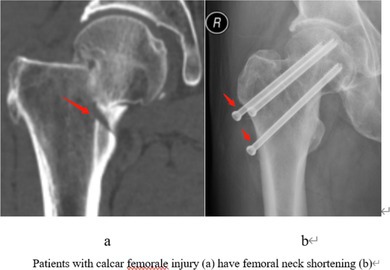

## 1 Introduction

Femoral neck fracture refers to the fracture from the femoral head to the base of the femoral neck, accounting for 3.6% of all fractures and 48%–54% of hip fractures [1]. This disease is more common in elderly people, mainly due to the common osteoporosis in this age group, which means that even minor external forces such as falls and sprains may lead to femoral neck fractures. In contrast, femoral neck fractures in young and middle‐aged people are often caused by high‐energy trauma due to their greater bone strength. Since Sven Johansson pioneered the closed reduction and cannulated screws fixation technique for the treatment of femoral neck fractures in 1932, this surgical method has become the preferred treatment for this disease in young and middle‐aged patients [2]. However, in elderly patients, arthroplasty is generally preferred over internal fixation with cannulated screws due to the substantially higher complication rates associated with the latter approach [3]. Patients may face a series of complications after cannulated screws fixation surgery, such as femoral neck shortening, femoral head necrosis, and bone nonunion. According to statistics, the incidence of femoral neck shortening is relatively high, reaching about 30%, the incidence of femoral head necrosis is about 14.3%, and the incidence of bone nonunion is relatively low, about 9.3% [4, 5, 6]. The occurrence of complications increases the reoperation rate of the cannulated screws fixation technique for femoral neck fractures [7]. Therefore, in recent years, research on postoperative complications of femoral neck fractures has become the focus of academic attention. For young and middle‐aged patients with femoral neck fractures, since their fractures are mostly caused by high‐energy trauma and the proportion of unstable fractures is higher, they are at increased risk of complications [8]. In addition, compared with the elderly, young and middle‐aged patients have higher expectations and requirements for preserving the femoral head and restoring lower limb function. Postoperative complications are an important factor affecting the prognosis of young and middle‐aged patients, and it is particularly important to clarify their risk factors.

Upadhyay et al. studied 102 patients with femoral neck fractures and found that posterior cortical comminution, poor reduction, and improper screw placement were risk factors for nonunion [9]. Konarski et al. found that displaced fractures were risk factors for femoral head necrosis after internal fixation of femoral neck fractures [10]. Zhao et al. found that fracture type, posteromedial cortical comminution, and reduction quality could be used to predict postoperative femoral neck shortening. In addition, BMD and body mass index (BMI) may also be risk factors for femoral neck shortening [11]. The calcar femorale is a longitudinal dense bone plate that protrudes from the posterior medial cortical bone at the junction of the femoral neck and shaft into the medullary cavity. It is wide at the top and narrow at the bottom. It has the functions of bearing compressive loads, transmitting the load of the femoral head, and strengthening the base of the femoral neck [12, 13]. Jin et al. believed that the displacement of the calcar femorale, degree of comminution, and varus angle of the femoral neck would affect the treatment effect of femoral neck fracture [14]. Biomechanical studies have demonstrated that the calcar femorale provides important structural support in the proximal femur, particularly bearing compressive stress and resisting varus stresses [13]. Its integrity is therefore essential for maintaining femoral neck stability and preventing failure after fracture fixation. When damaged, the calcar femorale's load‐sharing capacity is compromised, potentially leading to increased fracture site micromotion and subsequent complications [15]. Previous studies have seldom explored the relationship between the calcar femorale and postoperative complications of femoral neck fractures. Therefore, the research objectives of this paper are as follows: (1) to clarify the imaging anatomical characteristics of the calcar femorale; (2) to observe the injury situation of the calcar femorale in femoral neck fractures; and (3) to explore the relationship between various factors such as the calcar femorale, sex, age, BMI, and postoperative complications of femoral neck fractures in young and middle‐aged patients.

## 2 Materials and Methods

### 2.1 General Information

A total of 350 patients with femoral neck fractures admitted to our hospital from 2015 to 2020 were selected, and the patients' sex, age, BMI, Garden classification (displaced and non‐displaced), calcar femorale injury, comorbidities such as hypertension, diabetes, coronary artery disease (CAD), and renal dysfunction (defined as Scr ≥ 1.5× upper limit of normal [ULN]), femoral neck region computed tomography Hounsfield units (CT HUs), postoperative complications (femoral neck shortening, nonunion, and femoral head necrosis) and other clinical and imaging information were collected. The STROBE checklist was used in the study.

### 2.2 Inclusion and Exclusion Criteria

Inclusion criteria: (1) All patients diagnosed with femoral neck fracture; (2) All patients underwent closed reduction and internal fixation with three cannulated screws; (3) Patients with good reduction quality (Restoration of the smooth S‐curve between the femoral head and neck on both anteroposterior and lateral views) [16]; (4) Aged between 18 and 65 years (this represents the definition of ‘young and middle‐aged’ populations [17]. Moreover, patients aged > 65 years are more likely to receive arthroplasty rather than internal fixation due to higher rates of complications [18]); (5) Follow‐up time exceeded 1 year. Exclusion criteria: (1) Patients with pathological fractures or old fractures (fracture time > 3 weeks); (2) Patients with multiple ipsilateral hip fractures; (3) Patients with severe hip osteoarthritis or hip deformity; (4) Patients with incomplete preoperative or postoperative imaging data.

### 2.3 Observation of Radiological Manifestations of Calcar Femorale

In the hip CT images, the position of the calcar femorale in the femoral neck was observed in the coronal, sagittal, and transverse planes. The calcar femorale was reconstructed in three dimensions by importing the CT images into Mimics software.

### 2.4 Evaluation of Calcar Femorale Injuries and Postoperative Complications

Calcar femorale integrity was assessed based on the patient's CT images. Fracture lines extending through the calcar femorale were classified as damaged, while those sparing this structure were classified as undamaged. Femoral neck shortening was measured by comparing the first anteroposterior hip X‐ray after surgery with the last follow‐up X‐ray, and the longest part of the screw tail exposed on the lateral cortex of the femur was selected as the measurement mark. Nonunion was defined as a fracture that still had a clear fracture line visible on X‐ray or CT 9 months after surgery and no signs of healing for 3 consecutive months [19]. The diagnosis of femoral head necrosis was based on the Ficat criteria [20].

### 2.5 Statistical Analysis

SPSS 27.0 statistical software (IBM Corp., Armonk, NY, USA) was used to analyze the above indicators, and univariate analysis was performed on possible influencing factors of femoral neck fracture complications, such as sex, age, BMI, Garden classification, and calcar femorale injury. Mann–Whitney *U* test was used for inter‐group comparison; Pearson's chi‐square test, Yates' continuity correction, and Fisher's exact test were used for enumeration data comparison. Indicators with *p* < 0.05 in univariate analysis were included in multivariate Logistic regression analysis, and *p* < 0.05 indicated that the difference was statistically significant.

## 3 Results

### 3.1 CT Imaging Anatomical Features of the Calcar Femorale and Its Three‐Dimensional Reconstruction Images

**FIGURE 1:**
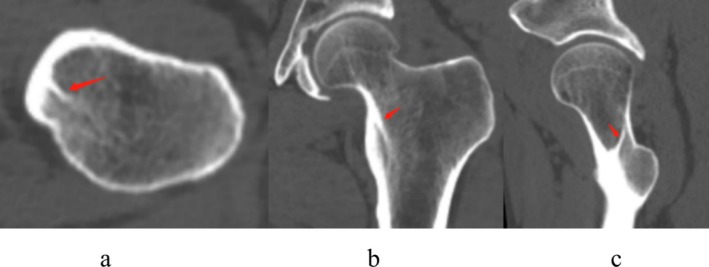
Multiplanar CT visualization of calcar femorale anatomy. (a) Axial view demonstrates the dense cortical projection (red arrow) extending from the posteromedial femoral cortex. (b) Coronal reconstruction shows the orientation of the calcar femorale (red arrow) along the femoral neck axis. (c) Sagittal view demonstrates the calcar femorale's anatomical continuity (red arrow), with its superior aspect merging with the mid‐femoral neck's posteromedial cortex and its inferior aspect blending with the lesser trochanter's cortical bone.

**FIGURE 2:**
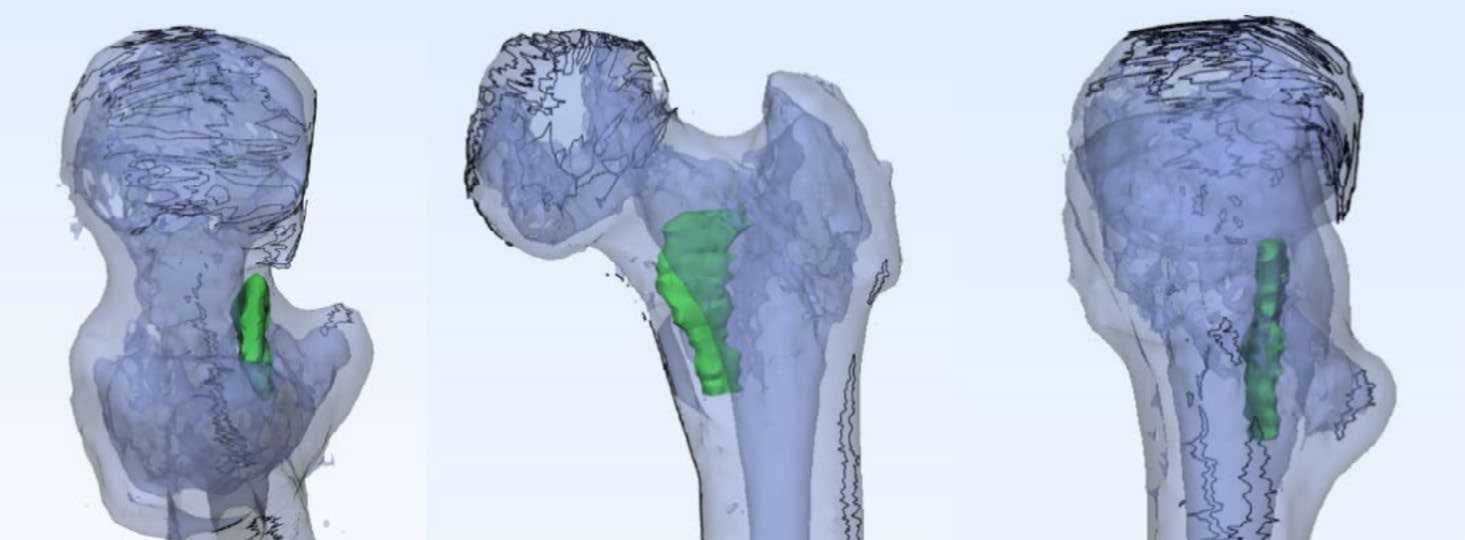
The green part is the 3D reconstructed calcar femorale.

Figure 1 displays the imaging features of the calcar femorale in CT scans, illustrating its appearance in cross‐sectional, coronal, and sagittal planes. On the other hand, Figure 2 presents a three‐dimensional reconstruction of the calcar femorale. From the images, it is evident that the calcar femorale comprises a dense bone plate that extends from the posteromedial cortex of the proximal femur into the cancellous bone. Notably, its upper and lower ends merge with the posteromedial cortex of the middle section of the femoral neck and the lesser trochanter, respectively.

### 3.2 The Relationship Between the Calcar Femorale and the Predilection Site of Femoral Neck Fracture

**FIGURE 3:**
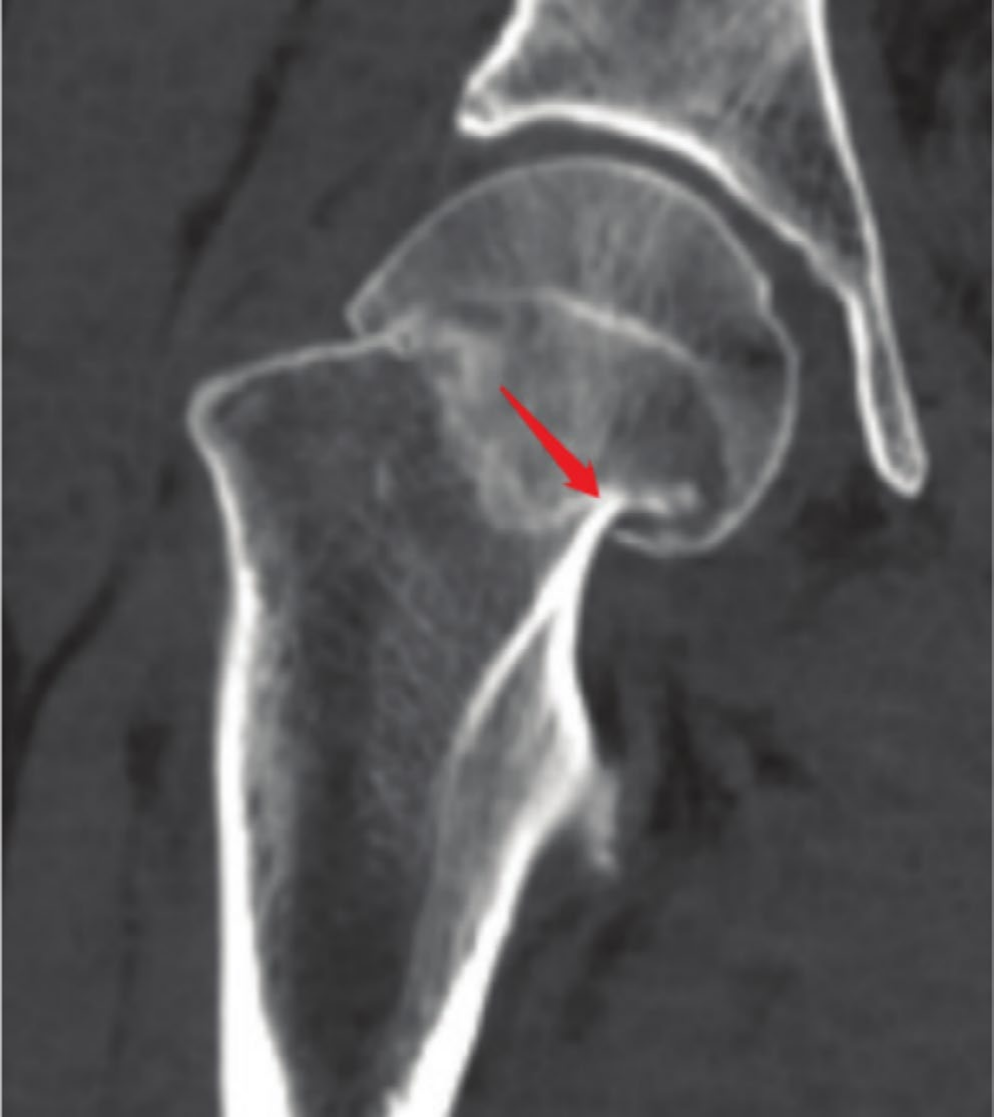
The junction between the calcar femorale and the posteromedial cortex and its proximal end are the predilection sites for femoral neck fractures (red arrows).

According to the inclusion and exclusion criteria, 284 patients were included for analysis, including 133 males and 151 females, with an average age of 49 years, a mean BMI of 22.77 kg/m^2^, 150 cases of calcar femorale injury, 134 cases of calcar femorale non‐injury, 115 cases of postoperative complications, 105 cases of femoral neck shortening, 12 cases of nonunion, 32 cases of femoral head necrosis, 135 cases of Garden classification non‐displaced type (Type I, II), and 149 cases of displaced type (Type III, IV). Among them, the fracture line passed through the junction of the calcar femorale and the posteromedial cortex and its proximal end in 235 cases (accounting for 82.7%) (Figure 3).

### 3.3 Univariate and Multivariate Analysis of Femoral Neck Shortening in Patients With Femoral Neck Fracture

**FIGURE 4:**
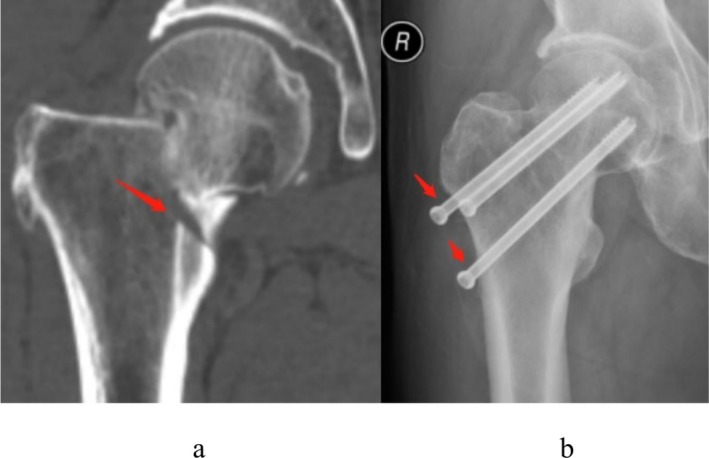
Radiographic findings in a femoral neck fracture case: (a) initial imaging demonstrating calcar femorale injury (red arrow); (b) subsequent screw migration due to femoral neck shortening (red arrows).

As can be seen from Table 1, in univariate analysis, sex, age, BMI, Garden classification, calcar femorale injury, and CT HUs showed significant differences between the two groups of whether shortening of femoral neck fracture occurred (*p* < 0.05). Multivariate logistic regression analysis shows that the Garden classification, the calcar femorale injury, and lower CT HUs were independent risk factors for femoral neck shortening, while age, sex, BMI, and comorbidities have nothing to do with femoral neck shortening, and the difference is not statistically significant (*p* > 0.05). Figure 4 shows the occurrence of femoral neck shortening in patients with calcar femorale injury.

**TABLE 1:** Univariate and multivariate analysis of femoral neck shortening in patients with femoral neck fracture.

Characteristics	Univariate analysis		Multivariate analysis		
	*χ*2/*Z*	*p*	Odds ratio	95% CI	*p*
Sex	9.985	**0.002**	0.705	0.368–1.352	0.293
Age	−2.281	**0.023**	1.021	0.991–1.051	0.179
Body mass index	−3.468	**< 0.001**	1.095	0.997–1.202	0.059
Garden classification	47.203	**< 0.001**	4.615	2.479–8.593	**< 0.001**
Calcar femorale injury	25.587	**< 0.001**	1.990	1.087–3.645	**0.026**
CT HUs	−4.714	< 0.001	0.989	0.982–0.996	**0.002**
Abnormal renal function	0.000	1.000	/	/	/
Hypertension	1.686	0.194	/	/	/
Diabetes	0.166	0.683	/	/	/
Coronary heart disease	0.000	1.000	/	/	/

*Note*: Bold values represent *p* < 0.05, indicating statistical differences.

### 3.4 Univariate and Multivariate Analysis of Nonunion in Patients With Femoral Neck Fracture

As can be seen from Table 2, in univariate analysis, sex, Garden classification, and whether the patient has diabetes showed significant differences (*p* < 0.05) among people with or without nonunion of femoral neck fractures. Multivariate analysis showed that only whether the patient has diabetes was an independent risk factor for nonunion of femoral neck fractures.

**TABLE 2:** Univariate and multivariate analysis of nonunion in patients with femoral neck fracture.

Characteristics	Univariate analysis		Multivariate analysis		
	*χ*2/*Z*	*p*	Odds ratio	95% CI	*p*
Sex	3.993	**0.046**	0.346	0.084–1.430	0.143
Age	−1.828	0.068	/	/	/
Body mass index	−1.514	0.130	/	/	/
Garden classification	7.721	**0.005**	8.167	0.999–66.748	0.050
Calcar femorale injury	0.964	0.326	/	/	/
CT HUs	−1.796	0.073	/	/	/
Abnormal renal function		0.122	/	/	/
Hypertension	0.934	0.334	/	/	/
Diabetes		**0.013**	10.069	2.043–49.628	**0.005**
Coronary heart disease		1.000	/	/	/

*Note*: Bold values represent *p* < 0.05, indicating statistical differences.

### 3.5 Univariate and Multivariate Analysis of Femoral Head Necrosis in Patients With Femoral Neck Fracture

As can be seen from Table 3, in univariate analysis, BMI and Garden classification showed significant differences between the two groups of people with or without femoral head necrosis after femoral neck fracture (*p* < 0.05). Multivariate analysis showed that BMI and Garden classification were independent risk factors for femoral head necrosis.

**TABLE 3:** Univariate and multivariate analysis of femoral head necrosis in patients with femoral neck fracture.

Characteristics	Univariate analysis		Multivariate analysis		
	*χ*2/*Z*	*p*	Odds ratio	95% CI	*p*
Sex	0.145	0.703	/	/	/
Age	−0.862	0.389	/	/	/
Body mass index	−2.854	**0.004**	1.154	1.014–1.313	**0.030**
Garden classification	21.057	**< 0.001**	10.000	2.950–33.903	**< 0.001**
Calcar femorale injury	1.357	0.244	/	/	/
CT HUs	−1.894	0.058	/	/	/
Abnormal renal function		0.302	/	/	/
Hypertension	2.381	0.123	/	/	/
Diabetes	0.001	0.975	/	/	/
Coronary heart disease		0.302	/	/	/

*Note*: Bold values represent *p* < 0.05, indicating statistical differences.

## 4 Discussion

The study revealed that the junction of the calcar femorale and the posteromedial cortex and its proximal end are predilection sites for femoral neck fractures. For young and middle‐aged patients with femoral neck fractures and calcar femorale injuries, the risk of postoperative femoral neck shortening is significantly increased. Its independent risk factors also include lower CT Hounsfield units and Garden classifications with displaced types. Diabetes is a risk factor for nonunion. Moreover, patients with higher BMI and displaced type fractures are more likely to develop femoral head necrosis after surgery. Therefore, clinicians need to attach great importance to the above risk factors in order to reduce postoperative complications and improve the prognosis of young and middle‐aged patients with femoral neck fractures.

### 4.1 Anatomical Characteristics and 3D Reconstruction of the Calcar Femorale

The calcar femorale is an approximately triangular (wide at the top and narrow at the bottom) longitudinal dense bone plate that protrudes from the medial cortical bone of the proximal femoral neck to the medullary cavity. Familiarity with its structure is important for the treatment of femoral neck fractures. The 3D reconstruction of the calcar femorale allows clinicians to visualize its anatomical morphology and precisely locate its position within the medullary cavity, thereby facilitating better surgical decision‐making to reduce complications. For instance, some studies suggest that DHS (dynamic hip screw) with a cannulated screw fixation may be more suitable for patients with calcar femorale involvement [14]. Additionally, understanding the 3D morphology of the calcar femorale is crucial for optimizing the design of fracture fixation and enhancing robotic‐assisted fracture reconstruction. Evidence indicates that fixation closer to the calcar femorale provides greater stability [21]. In the future, robot‐assisted technology integrated with 3D reconstruction may facilitate more accurate screw placement near the calcar femorale, thereby enhancing post‐operative stability, further reducing complications like femoral neck shortening [22].

### 4.2 Predilection Site of Femoral Neck Fracture

The finding that 82.7% of femoral neck fractures occur at the junction and its proximal end of the calcar femorale and posterior medial cortex. This area represents a biomechanical “weak point” where stress concentration occurs during trauma. Therefore, strengthening the fixation of the weak point is beneficial for reducing postoperative complications. McGarry et al.'s related research shows that strengthening medial cortical support of the femoral neck fractures exhibits good clinical outcomes with low rates of failure and complications [23].

### 4.3 Risk Factors for Femoral Neck Shortening

A retrospective study by Stockton et al. found that 32% of young people experienced femoral neck shortening after femoral neck fracture surgery, which is a worrying finding [6]. Since young and middle‐aged people have a higher demand for hip preservation, multiple parallel hollow compression screws are the main treatment method in clinical practice. The characteristic of this internal fixation device is that it allows sliding compression between fracture fragments to promote fracture healing. However, the sliding compression between the internal fixation device and the fracture end is also the cause of femoral neck shortening after surgery [24]. Sliding compression promotes bone absorption at the femoral neck fracture end, resulting in femoral neck shortening. Our findings regarding calcar femorale injuries in femoral neck shortening align well with the established biomechanical role of the calcar femorale. As a dense cortical strut, the intact calcar femorale serves as an internal buttress that resists compressive forces across the femoral neck [13, 15]. When this structure is disrupted by fracture, the remaining fixation constructs must bear greater loads. This explains why patients with calcar femorale injuries in our study demonstrated significantly higher rates of femoral neck shortening. Polat et al. divided 81 patients after internal fixation of femoral neck fractures into two groups according to the presence or absence of femoral neck shortening. They used a retrospective study method to analyze the independent risk factors for postoperative femoral neck shortening. The average age of the patients was 42.2 ± 17.8 years old; significant differences were found between the two groups in terms of Garden classification and whether the medial femoral neck was supported by a plate [25]. Polat's research shows that the mechanical support on the medial side of the femoral neck affects the occurrence of shortening. Garden III and IV types are often accompanied by partial comminuted fractures and posterior cortical destruction, and the bone absorption is more severe. The results of this study are similar. The calcar femorale is a dense bone plate formed by the posteromedial cortical bone at the junction of the femoral neck shaft. Our study found that lower CT HUs, calcar femorale injury, and Garden classification as a displaced type are potential independent risk factors for femoral neck shortening. The CT HUs reflect the bone mineral density [26]. In patients with low bone density, the screw fixation holding force is compromised after internal fixation, accompanied by a reduction in the internal fixation material's capacity to resist axial compression at the fracture site. After surgery, the bone tissue at the fracture end is more susceptible to resorption, thereby increasing the risk of femoral neck shortening under compressive forces [27]. These findings hold significant clinical relevance for mitigating femoral neck shortening. Specifically, for patients presenting with marked fracture displacement (Garden III/IV), calcar femorale injury, or low bone density (evidenced by reduced CT HUs), the use of augmented fixation constructs (e.g., DHS with anti‐rotational screw or medial buttress plating) is recommended to enhance biomechanical stability.

### 4.4 Risk Factors for Nonunion

In addition to femoral neck shortening, nonunion is also a major complication of femoral neck fractures. Several studies have identified multiple risk factors for nonunion, including high Pauwels grade, inadequate fracture reduction, and suboptimal fixation methods [26, 28, 29]. Our multivariate logistic regression analysis further established diabetes mellitus as an independent predictor of nonunion, corroborating existing evidence on diabetes‐associated impairments in bone healing mechanisms [30, 31]. The detrimental effects of diabetes on bone metabolism are multifactorial, mainly including excessive advanced glycation end products, increased reactive oxygen species, macrophage polarization disorder, microvascular system deterioration, and immune imbalance in diabetes patients. This will lead to a disruption of the balance between osteoblasts and osteoclasts, resulting in reduced bone formation and an increased risk of bone nonunion or delayed healing [30, 32]. These findings emphasize the necessity of cooperation with endocrinologists in perioperative management. In addition, diabetes patients need closer radiological follow‐up to find early signs of nonunion.

### 4.5 Risk Factors for Femoral Head Necrosis

Femoral head necrosis is also an important complication. Ju et al. conducted a retrospective study on 297 patients with femoral neck fractures treated with internal fixation and classified the patients according to the Garden classification. It was found that there was a significant difference in the femoral head necrosis rate between different groups, which was similar to the results of this study [33]. It may be because the Garden classification represents the degree of displacement. The greater the degree of displacement, to a certain extent, the greater the degree of trauma at the time of injury, which can cause more serious damage to local microvessels, trabeculae, and other fine structures, resulting in a slower post‐operative blood supply reconstruction [33]. Pei et al. conducted a retrospective analysis of 250 patients under the age of 60 with femoral neck fractures and found that BMI > 25 was an independent risk factor for femoral head necrosis after femoral neck surgery, which is consistent with the results of this study [34]. BMI is closely related to the total amount of body fat. Its increase increases the load on the hip joint and may have a negative impact on the reconstruction of femoral head blood supply. On the other hand, the increased blood viscosity in overweight patients will cause blood flow retardation and affect the microcirculation blood supply of the femoral head [35]. Based on the above risk factor, personalized treatment strategies can be developed clinically. For high‐risk patients with femoral head necrosis (such as Garden classification with displaced type combined with obesity), more proactive intervention measures may need to be prioritized, such as early capsulotomy or revascularization surgery to promote postoperative blood supply recovery [36, 37].

### 4.6 Strengths and Limitations

The advantage of this study is that it is the first to report the relationship between calcar femorale injury and complications, and the sample size is large, which provides a reference for reducing the occurrence of postoperative complications in the clinical diagnosis and treatment of femoral neck fractures. Moreover, by highlighting the significance of calcar femorale injury and other risk factors, our findings can guide clinical doctors to closely monitor patients or design better internal fixation devices and surgical methods to reduce the incidence of postoperative complications. However, this study is a retrospective study, and there is inevitably a bias in the admission rate, which has a certain impact on the authenticity of the results. It is hoped that the relationship between calcar femorale injury and complications can be further confirmed in future multicenter, large‐sample randomized controlled studies, providing a more feasible theoretical basis for clinical diagnosis and treatment.

## 5 Conclusion

This study elucidates the anatomical features of the calcar femorale and highlights the critical role of calcar femorale integrity in postoperative stability and identifies key risk factors (e.g., calcar femorale injury, displaced fracture patterns (Garden III/IV), low CT Hounsfield units, diabetes mellitus, and elevated BMI). These findings advocate for tailored interventional strategies to improve outcomes in young and middle‐aged patients with femoral neck fractures. Future prospective or biomechanical studies could further validate these findings and refine surgical approaches.

## Author Contributions

J.W. designed this study. Q.L. and K.Z. wrote the manuscript. B.Z. analyzed and interpreted the data. T.Z. and W.H. analyzed the data and prepared all the figures. H.D. provided technical support. All authors read and approved the final manuscript.

## Disclosure

All authors listed meet the authorship criteria according to the latest guidelines of the International Committee of Medical Journal Editors, and all authors are in agreement with the manuscript.

## Ethics Statement

Ethical approval was obtained by the Medical Ethics Committee of Beijing Jishuitan Hospital (No. 202203‐100). The informed consent was obtained from all subjects and/or their legal guardian(s). All methods were carried out in accordance with relevant guidelines and regulations.

## Conflicts of Interest

The authors declare no conflicts of interest.

## Data Availability

The data that support the findings of this study are available from the corresponding author upon reasonable request.
